# *cis*-*cis*-*trans*-Bis(acetonitrile-κ*N*)dichloridobis(triphenyl­phosphine-κ*P*)ruthenium(II) acetonitrile disolvate

**DOI:** 10.1107/S1600536807065968

**Published:** 2007-12-12

**Authors:** Ahmad M. Al-Far, LeGrande M. Slaughter

**Affiliations:** a Department of Chemistry, Oklahoma State University, Stillwater, OK 74078, USA

## Abstract

The title compound, [RuCl_2_(C_2_H_3_N)_2_(C_18_H_15_P)_2_]·2C_2_H_3_N, was obtained upon stirring an acetonitrile/ethanol solution of [RuCl_2_(PPh_3_)_3_]. In the crystal structure, each Ru^II^ ion is coordinated by two Cl [Ru—Cl = 2.4308 (7) and 2.4139 (7) Å], two N [Ru—N = 2.016 (2) and 2.003 (2) Å], and two P [Ru—P = 2.3688 (7) and 2.3887 (7) Å] atoms in a distorted octa­hedral geometry. Packing inter­actions include typical C—H⋯π contacts involving phenyl groups as well as weak hydrogen bonds between CH_3_CN methyl H atoms and Cl or solvent CH_3_CN N atoms.

## Related literature

For the original synthesis, characterization and reactivity of the title compound and its precursor, see: Gilbert & Wilkinson (1969 ▶); Stephenson & Wilkinson (1966 ▶); Hallman *et al.* (1970 ▶); Caulton (1974 ▶).
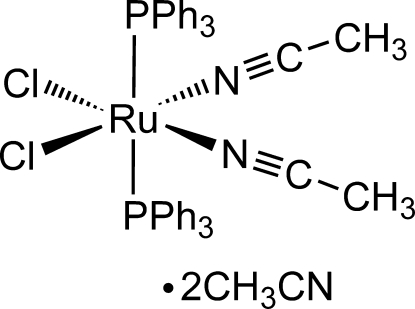


## Experimental

### 

#### Crystal data

[RuCl_2_(C_2_H_3_N)_2_(C_18_H_15_P)_2_]·2C_2_H_3_N
*M* *_r_* = 860.73
Orthorhombic, 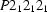

*a* = 9.0622 (9) Å
*b* = 18.0167 (18) Å
*c* = 25.628 (2) Å
*V* = 4184.3 (7) Å^3^
*Z* = 4
Mo *K*α radiation
μ = 0.61 mm^−1^
*T* = 170 (2) K
0.40 × 0.35 × 0.20 mm

#### Data collection

Bruker SMART APEXII CCD diffractometer
Absorption correction: multi-scan (*SADABS*; Sheldrick, 2000 ▶) *T*_min_ = 0.791, *T*_max_ = 0.887
25910 measured reflections
10568 independent reflections
9200 reflections with *I* > 2σ(*I*)
*R*_int_ = 0.042

#### Refinement

*R*[*F* ^2^ > 2σ(*F* ^2^)] = 0.035
*wR*(*F* ^2^) = 0.072
*S* = 1.02
10568 reflections
482 parameters
H-atom parameters constrained
Δρ_max_ = 0.38 e Å^−3^
Δρ_min_ = −0.30 e Å^−3^
Absolute structure: Flack (1983 ▶), 4387 Friedel pairs
Flack parameter: −0.02 (2)

### 

Data collection: *APEX2* (Bruker, 2006 ▶); cell refinement: *SAINT* (Bruker, 2006 ▶); data reduction: *SAINT*; program(s) used to solve structure: *SHELXTL* (Sheldrick, 2000 ▶); program(s) used to refine structure: *SHELXTL*; molecular graphics: *SHELXTL*; software used to prepare material for publication: *SHELXTL*.

## Supplementary Material

Crystal structure: contains datablocks global, I. DOI: 10.1107/S1600536807065968/ci2537sup1.cif

Structure factors: contains datablocks I. DOI: 10.1107/S1600536807065968/ci2537Isup2.hkl

Additional supplementary materials:  crystallographic information; 3D view; checkCIF report

## Figures and Tables

**Table 1:** Hydrogen-bond geometry (Å, °) *Cg*1 is the centroid of the C51–C56 phenyl ring.

*D*—H⋯*A*	*D*—H	H⋯*A*	*D*⋯*A*	*D*—H⋯*A*
C4—H4*B*⋯Cl1^i^	0.98	2.68	3.560 (3)	149
C101—H101⋯Cl1^ii^	0.98	2.80	3.698 (4)	153
C2—H2*C*⋯Cl2^iii^	0.98	2.57	3.544 (3)	175
C101—H102⋯Cl2	0.98	2.62	3.554 (4)	158
C2—H2*A*⋯N100^i^	0.98	2.60	3.519 (5)	155
C101—H103⋯N200	0.98	2.72	3.645 (6)	158
C201—H201⋯N200^iv^	0.98	2.66	3.526 (7)	148
C64—H64⋯*Cg*1^iii^	0.95	2.96	3.715 (4)	138

Symmetry codes: (i) 
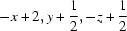
; (ii) 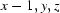; (iii) 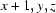; (iv) 
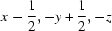
.

**Fig. 1.**
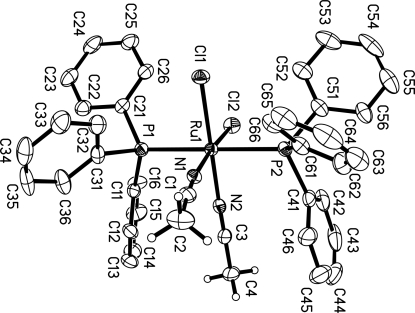
ORTEP view of the complex portion of the title compound, with displacement ellipsoids at the 50% probability level. Phenyl hydrogen atoms and acetonitriles of crystallization are omitted for clarity.

**Fig. 2.**
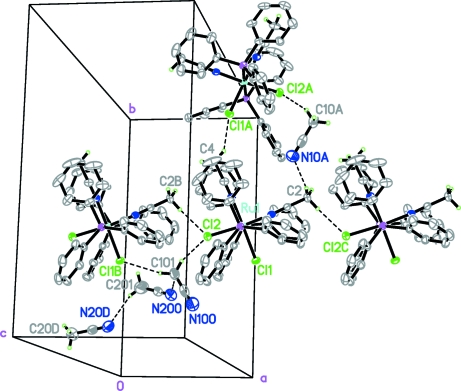
Packing diagram showing a portion of the network of weak hydrogen bonds involving acetonitrile C—H bonds. Symmetry codes: (A) 2 - x, 1/2 + y, 1/2 - z; (B) -1 + x, y, z; (C) 1 + x, y, z; (D) -1/2 + x, 1/2 - y, -z. For solvent symmetry equivalents, N200 becomes N20A, etc.

## supplementary crystallographic information

### Comment

[RuCl_2_(PPh_3_)_3_] has been widely used as a convenient synthon for a variety of Ru^II^ complexes (Stephenson & Wilkinson, 1966; Hallman *et al.*, 1970). It readily loses one phosphine ligand in solution to give solvent adducts or chlorido-bridged Ru^II^ species that are potential catalyst precursors (Caulton, 1974). Gilbert & Wilkinson (1969) previously reported the synthesis of two isomers of [RuCl_2_(CH_3_CN)_2_(PPh_3_)_2_] having either *cis* or *trans* orientations of the acetonitrile ligands as characterized by infrared spectroscopy. The *cis* isomer was obtained upon refluxing [RuCl_2_(PPh_3_)_3_] in CH_3_CN/acetone, whereas the *trans* isomer was formed upon refluxing in CH_3_CN/toluene. We found that the *cis* isomer could also be obtained by stirring [RuCl_2_(PPh_3_)_3_] in CH_3_CN/ethanol at ambient temperature, confirming the importance of a polar co-solvent in favoring a *cis* geometry.

The crystal structure of the title compound contains one [RuCl_2_(CH_3_CN)_2_(PPh_3_)_2_] complex and two acetonitriles of crystallization in the asymmetric unit. The Ru^II^ complex displays a *cis* orientation of both the chlorido and CH_3_CN ligands and a *trans* orientation of the phosphine ligands (Fig. 1). The Ru—Cl [2.4308 (7), 2.4139 (7) Å], Ru—N [2.016 (2), 2.003 (2) Å], and Ru—P [2.3688 (7), 2.3887 (7) Å] distances are in the expected ranges, and the angles between coordinated atoms are in the range 90.02 (6)—93.83 (2)°. In addition to typical C—H···π packing interactions involving phenyl rings, there are several weak hydrogren bonds between C—H bonds of coordinated or solvate acetonitriles and Cl ligands or solvate acetonitrile N atoms (Fig. 2). The H···acceptor distances range from 2.57—2.80 Å, and the C···acceptor distances range from 3.52—3.70 Å (Table 1).

Although it has been little investigated, [RuCl_2_(CH_3_CN)_2_(PPh_3_)_2_] is a potentially useful precursor for catalytically active Ru species given the presence of two dissociable ligands in a *cis* arrangement.

### Experimental

[RuCl_2_(PPh_3_)_3_] (20 mg) was dissolved in a mixture of degassed absolute ethanol (2 ml) and freshly distilled CH_3_CN (3 ml) and stirred for 15 min. During this time, the color of the solution changed from dark brown to yellow. The solvent was removed under vacuum, and the resulting yellow powder was dried for a further 2 h. A 10 mg portion of the solid was dissolved in 0.6 ml of acetonitrile and alllowed to stand for 3 d under nitrogen. Large yellow-orange crystals of the title compound formed over this time. The crystals became opaque due to solvent loss within 20 min of removal from acetonitrile unless placed in a cold stream. The sample used in this study was cut from a larger (>1 mm) block, immersed in Paratone N oil in a 0.5 mm nylon loop, and placed in the nitrogen cold stream of an APEXII diffractometer at 170 (2) K for X-ray diffraction analysis.

### Refinement

Phenyl H atoms were fixed at C—H distances of 0.95 Å and refined as riding, with *U*_iso_(H) = 1.2*U*_eq_(C). Methyl H atoms were placed with idealized threefold symmetry and fixed C—H distances of 0.98 Å, and they were refined in a riding model with *U*_iso_(H) = 1.5*U*_eq_(C). In order to assign the absolute structure, 4387 Friedel pairs (71% of all Friedel pairs) were measured, and Friedel opposites were not merged in the reflection list used for structure solution and refinement. The absolute structure parameter (Flack *x*) refined to -0.02 (2). For the inverted structure, Flack *x* refined to 1.02 (2), and increases in *R*[*F*^2^>2σ(*F*^2^)] and *wR*(*F*^2^) of 0.33% and 1.29%, respectively, were observed.

### Crystal data

**Table:**

[RuCl_2_(C_2_H_3_N)_2_(C_18_H_15_P)_2_]·2C_2_H_3_N	*F*_000_ = 1768
*M**_r_* = 860.73	*D*_x_ = 1.366 Mg m^−^^3^
Orthorhombic, *P*2_1_2_1_2_1_	Mo *K*α radiation λ = 0.71073 Å
Hall symbol: P 2ac 2ab	Cell parameters from 7584 reflections
*a* = 9.0622 (9) Å	θ = 2.6–29.0º
*b* = 18.0167 (18) Å	µ = 0.61 mm^−^^1^
*c* = 25.628 (2) Å	*T* = 170 (2) K
*V* = 4184.3 (7) Å^3^	Block, orange
*Z* = 4	0.40 × 0.35 × 0.20 mm

### Data collection

**Table:**

Bruker SMART APEXII CCD diffractometer	10568 independent reflections
Radiation source: fine-focus sealed tube	9200 reflections with *I* > 2σ(*I*)
Monochromator: graphite	*R*_int_ = 0.042
Detector resolution: 0.75 pixels mm^-1^	θ_max_ = 29.0º
*T* = 170(2) K	θ_min_ = 2.0º
φ and ω scans	*h* = −12→11
Absorption correction: multi-scan(SADABS; Sheldrick, 2000)	*k* = −23→24
*T*_min_ = 0.791, *T*_max_ = 0.887	*l* = −24→34
25910 measured reflections	

### Refinement

**Table:**

Refinement on *F*^2^	*w* = 1/[σ^2^(*F*_o_^2^) + (0.0197*P*)^2^ + 1.2863*P*] where *P* = (*F*_o_^2^ + 2*F*_c_^2^)/3
Least-squares matrix: full	(Δ/σ)_max_ = 0.002
*R*[*F*^2^ > 2σ(*F*^2^)] = 0.035	Δρ_max_ = 0.38 e Å^−^^3^
*wR*(*F*^2^) = 0.072	Δρ_min_ = −0.30 e Å^−^^3^
*S* = 1.02	Extinction correction: none
10568 reflections	Absolute structure: Flack (1983), 4387 Friedel pairs
482 parameters	Flack parameter: −0.02 (2)
H-atom parameters constrained	

### Special details

**Table:**

Geometry. All e.s.d.'s (except the e.s.d. in the dihedral angle between two l.s. planes) are estimated using the full covariance matrix. The cell e.s.d.'s are taken into account individually in the estimation of e.s.d.'s in distances, angles and torsion angles; correlations between e.s.d.'s in cell parameters are only used when they are defined by crystal symmetry. An approximate (isotropic) treatment of cell e.s.d.'s is used for estimating e.s.d.'s involving l.s. planes.
Refinement. Refinement of *F*^2^ against ALL reflections. The weighted *R*-factor *wR* and goodness of fit *S* are based on *F*^2^, conventional *R*-factors *R* are based on *F*, with *F* set to zero for negative *F*^2^. The threshold expression of *F*^2^ > σ(*F*^2^) is used only for calculating *R*-factors(gt) *etc*. and is not relevant to the choice of reflections for refinement. *R*-factors based on *F*^2^ are statistically about twice as large as those based on *F*, and *R*- factors based on ALL data will be even larger.

### Fractional atomic coordinates and isotropic or equivalent isotropic displacement parameters (Å^2^)

**Table:**

	*x*	*y*	*z*	*U*_iso_*/*U*_eq_	
Ru1	0.98665 (2)	0.525404 (10)	0.202447 (8)	0.01835 (5)	
N1	1.1935 (2)	0.56649 (11)	0.20310 (9)	0.0221 (4)	
C1	1.3072 (3)	0.59216 (14)	0.20769 (11)	0.0260 (6)	
C2	1.4541 (3)	0.62345 (18)	0.21633 (14)	0.0424 (9)	
H2A	1.4572	0.6478	0.2505	0.064*	
H2B	1.4759	0.6599	0.1890	0.064*	
H2C	1.5278	0.5836	0.2152	0.064*	
N2	0.9070 (2)	0.62612 (12)	0.21976 (8)	0.0220 (5)	
C3	0.8554 (3)	0.68174 (16)	0.22992 (11)	0.0268 (6)	
C4	0.7865 (4)	0.75235 (16)	0.24243 (12)	0.0371 (7)	
H4A	0.7477	0.7749	0.2105	0.056*	
H4B	0.8598	0.7855	0.2581	0.056*	
H4C	0.7055	0.7442	0.2671	0.056*	
Cl1	1.07873 (8)	0.40246 (4)	0.18086 (3)	0.02873 (15)	
Cl2	0.73218 (6)	0.48601 (4)	0.20527 (3)	0.02807 (14)	
P1	0.95830 (7)	0.55102 (4)	0.11234 (3)	0.02106 (14)	
P2	1.00686 (7)	0.49955 (3)	0.29358 (2)	0.02023 (12)	
C11	0.8461 (3)	0.63406 (16)	0.10137 (10)	0.0234 (6)	
C12	0.9038 (4)	0.70435 (16)	0.11118 (11)	0.0311 (7)	
H12	1.0054	0.7095	0.1195	0.037*	
C13	0.8153 (4)	0.76658 (17)	0.10896 (13)	0.0392 (8)	
H13	0.8569	0.8141	0.1155	0.047*	
C14	0.6677 (4)	0.76067 (19)	0.09743 (13)	0.0409 (8)	
H14	0.6073	0.8037	0.0957	0.049*	
C15	0.6078 (4)	0.69085 (19)	0.08828 (14)	0.0426 (8)	
H15	0.5058	0.6861	0.0805	0.051*	
C16	0.6965 (3)	0.62787 (17)	0.09043 (12)	0.0321 (7)	
H16	0.6545	0.5803	0.0844	0.039*	
C21	0.8672 (3)	0.48398 (16)	0.06914 (10)	0.0235 (6)	
C22	0.8493 (3)	0.50217 (17)	0.01617 (11)	0.0304 (7)	
H22	0.8862	0.5480	0.0033	0.036*	
C23	0.7778 (4)	0.45353 (19)	−0.01746 (12)	0.0376 (7)	
H23	0.7652	0.4664	−0.0531	0.045*	
C24	0.7249 (4)	0.3863 (2)	0.00102 (13)	0.0407 (8)	
H24	0.6764	0.3529	−0.0219	0.049*	
C25	0.7430 (3)	0.36848 (18)	0.05247 (12)	0.0367 (7)	
H25	0.7059	0.3225	0.0650	0.044*	
C26	0.8147 (3)	0.41607 (16)	0.08707 (12)	0.0302 (7)	
H26	0.8276	0.4023	0.1225	0.036*	
C31	1.1296 (3)	0.56553 (17)	0.07510 (11)	0.0274 (6)	
C32	1.2373 (3)	0.51060 (18)	0.07986 (12)	0.0356 (7)	
H32	1.2235	0.4713	0.1041	0.043*	
C33	1.3649 (4)	0.5126 (2)	0.04960 (13)	0.0429 (8)	
H33	1.4369	0.4745	0.0529	0.052*	
C34	1.3864 (4)	0.5699 (2)	0.01487 (13)	0.0474 (9)	
H34	1.4726	0.5711	−0.0062	0.057*	
C35	1.2835 (4)	0.6250 (2)	0.01071 (13)	0.0498 (10)	
H35	1.3005	0.6652	−0.0125	0.060*	
C36	1.1541 (4)	0.6232 (2)	0.03999 (12)	0.0408 (8)	
H36	1.0825	0.6613	0.0360	0.049*	
C41	0.9408 (3)	0.57923 (16)	0.33105 (10)	0.0287 (7)	
C42	0.7897 (4)	0.5863 (2)	0.34125 (12)	0.0411 (8)	
H42	0.7238	0.5470	0.3331	0.049*	
C43	0.7367 (5)	0.6519 (2)	0.36353 (14)	0.0612 (13)	
H43	0.6346	0.6567	0.3714	0.073*	
C44	0.8310 (7)	0.7095 (2)	0.37417 (15)	0.0737 (16)	
H44	0.7939	0.7540	0.3892	0.088*	
C45	0.9794 (7)	0.7031 (2)	0.36316 (14)	0.0654 (13)	
H45	1.0441	0.7432	0.3705	0.078*	
C46	1.0343 (4)	0.63864 (18)	0.34158 (12)	0.0433 (9)	
H46	1.1365	0.6348	0.3339	0.052*	
C51	0.9060 (3)	0.42064 (16)	0.32141 (12)	0.0267 (6)	
C52	0.8560 (3)	0.36297 (16)	0.28975 (13)	0.0337 (7)	
H52	0.8711	0.3653	0.2531	0.040*	
C53	0.7839 (3)	0.30185 (18)	0.31140 (17)	0.0461 (10)	
H53	0.7493	0.2630	0.2895	0.055*	
C54	0.7629 (4)	0.2979 (2)	0.36451 (18)	0.0509 (11)	
H54	0.7147	0.2560	0.3792	0.061*	
C55	0.8113 (4)	0.3542 (2)	0.39642 (15)	0.0464 (9)	
H55	0.7962	0.3509	0.4330	0.056*	
C56	0.8823 (3)	0.41577 (18)	0.37543 (12)	0.0338 (7)	
H56	0.9148	0.4546	0.3977	0.041*	
C61	1.1917 (3)	0.48226 (16)	0.32086 (11)	0.0266 (6)	
C62	1.2245 (3)	0.49623 (18)	0.37348 (12)	0.0370 (7)	
H62	1.1517	0.5174	0.3956	0.044*	
C63	1.3620 (3)	0.4794 (2)	0.39327 (14)	0.0445 (8)	
H63	1.3825	0.4880	0.4291	0.053*	
C64	1.4698 (4)	0.45017 (19)	0.36158 (16)	0.0490 (9)	
H64	1.5650	0.4398	0.3753	0.059*	
C65	1.4394 (3)	0.43590 (18)	0.30962 (15)	0.0429 (9)	
H65	1.5135	0.4156	0.2876	0.051*	
C66	1.3001 (3)	0.45142 (16)	0.28976 (13)	0.0314 (7)	
H66	1.2791	0.4406	0.2542	0.038*	
N100	0.6221 (4)	0.2438 (2)	0.17890 (18)	0.0832 (13)	
C100	0.5542 (4)	0.2940 (3)	0.16867 (16)	0.0563 (11)	
C101	0.4699 (4)	0.3596 (2)	0.15297 (16)	0.0562 (10)	
H103	0.4561	0.3593	0.1150	0.084*	
H102	0.5237	0.4045	0.1632	0.084*	
H101	0.3734	0.3591	0.1702	0.084*	
N200	0.3740 (5)	0.3076 (2)	0.01978 (17)	0.0726 (11)	
C200	0.2643 (5)	0.3231 (2)	0.00348 (18)	0.0559 (11)	
C201	0.1228 (6)	0.3429 (3)	−0.0176 (3)	0.113 (2)	
H203	0.1257	0.3389	−0.0558	0.169*	
H202	0.0988	0.3940	−0.0077	0.169*	
H201	0.0473	0.3092	−0.0038	0.169*	

### Atomic displacement parameters (Å^2^)

**Table:**

	*U*^11^	*U*^22^	*U*^33^	*U*^12^	*U*^13^	*U*^23^
Ru1	0.01907 (9)	0.01683 (8)	0.01915 (9)	0.00050 (8)	−0.00009 (8)	0.00030 (9)
N1	0.0268 (11)	0.0175 (10)	0.0219 (11)	0.0018 (8)	0.0020 (11)	0.0027 (11)
C1	0.0260 (13)	0.0215 (13)	0.0304 (15)	0.0033 (10)	0.0048 (12)	0.0006 (14)
C2	0.0214 (15)	0.0347 (16)	0.071 (2)	−0.0042 (12)	0.0039 (14)	−0.0045 (17)
N2	0.0266 (12)	0.0218 (11)	0.0176 (11)	0.0005 (9)	−0.0019 (9)	0.0036 (10)
C3	0.0361 (16)	0.0250 (15)	0.0192 (14)	0.0006 (12)	−0.0029 (12)	0.0004 (13)
C4	0.058 (2)	0.0217 (15)	0.0312 (16)	0.0095 (14)	−0.0018 (14)	−0.0020 (13)
Cl1	0.0313 (4)	0.0206 (3)	0.0342 (4)	0.0044 (3)	0.0042 (3)	−0.0014 (3)
Cl2	0.0233 (3)	0.0293 (3)	0.0316 (3)	−0.0013 (2)	−0.0012 (3)	−0.0008 (3)
P1	0.0222 (3)	0.0215 (3)	0.0195 (3)	−0.0017 (3)	−0.0008 (3)	−0.0003 (3)
P2	0.0188 (3)	0.0210 (3)	0.0209 (3)	0.0012 (2)	0.0001 (3)	0.0029 (3)
C11	0.0292 (15)	0.0234 (14)	0.0176 (13)	−0.0022 (11)	−0.0036 (11)	0.0013 (12)
C12	0.0438 (18)	0.0250 (15)	0.0245 (15)	−0.0099 (13)	−0.0056 (13)	0.0047 (13)
C13	0.067 (2)	0.0201 (14)	0.0305 (16)	−0.0038 (16)	−0.0063 (15)	0.0059 (14)
C14	0.057 (2)	0.0299 (17)	0.0356 (18)	0.0124 (16)	−0.0032 (15)	0.0021 (16)
C15	0.0358 (19)	0.0411 (19)	0.051 (2)	0.0057 (15)	−0.0049 (15)	−0.0085 (18)
C16	0.0297 (16)	0.0246 (15)	0.0421 (18)	0.0022 (12)	−0.0026 (13)	−0.0073 (14)
C21	0.0220 (13)	0.0255 (15)	0.0229 (13)	0.0007 (11)	0.0003 (10)	−0.0039 (12)
C22	0.0348 (16)	0.0285 (15)	0.0278 (15)	0.0031 (12)	−0.0038 (12)	−0.0016 (13)
C23	0.0430 (18)	0.0436 (19)	0.0262 (15)	0.0044 (15)	−0.0070 (13)	−0.0106 (15)
C24	0.0390 (19)	0.047 (2)	0.0360 (18)	−0.0087 (16)	−0.0020 (15)	−0.0214 (17)
C25	0.0400 (18)	0.0325 (17)	0.0377 (18)	−0.0120 (14)	0.0066 (14)	−0.0113 (15)
C26	0.0353 (17)	0.0288 (16)	0.0265 (15)	−0.0020 (13)	0.0026 (12)	−0.0057 (13)
C31	0.0253 (15)	0.0366 (17)	0.0204 (13)	−0.0082 (12)	0.0006 (11)	0.0014 (13)
C32	0.0330 (16)	0.0366 (18)	0.0371 (17)	−0.0018 (13)	0.0112 (13)	−0.0009 (15)
C33	0.0334 (17)	0.052 (2)	0.0434 (19)	0.0000 (15)	0.0108 (14)	−0.0045 (18)
C34	0.0332 (19)	0.079 (3)	0.0303 (17)	−0.0140 (19)	0.0083 (14)	−0.0010 (19)
C35	0.043 (2)	0.079 (3)	0.0277 (17)	−0.015 (2)	0.0050 (15)	0.0214 (19)
C36	0.0340 (17)	0.060 (2)	0.0289 (16)	−0.0080 (16)	−0.0044 (13)	0.0162 (17)
C41	0.0408 (17)	0.0272 (15)	0.0182 (13)	0.0104 (12)	−0.0003 (11)	0.0039 (12)
C42	0.050 (2)	0.045 (2)	0.0290 (16)	0.0171 (16)	0.0111 (15)	0.0141 (16)
C43	0.085 (3)	0.066 (3)	0.0331 (19)	0.047 (2)	0.029 (2)	0.023 (2)
C44	0.153 (5)	0.043 (2)	0.026 (2)	0.044 (3)	0.003 (3)	−0.0040 (18)
C45	0.124 (4)	0.0335 (18)	0.0388 (19)	0.017 (3)	−0.025 (3)	−0.0080 (16)
C46	0.063 (2)	0.0347 (17)	0.0319 (16)	0.0037 (16)	−0.0126 (16)	−0.0022 (14)
C51	0.0171 (13)	0.0287 (15)	0.0345 (16)	0.0022 (11)	−0.0015 (11)	0.0107 (13)
C52	0.0248 (14)	0.0306 (15)	0.0458 (19)	0.0031 (11)	−0.0007 (14)	0.0083 (16)
C53	0.0288 (17)	0.0316 (17)	0.078 (3)	−0.0054 (13)	−0.0054 (17)	0.0155 (19)
C54	0.0262 (17)	0.043 (2)	0.083 (3)	0.0007 (15)	0.0091 (18)	0.039 (2)
C55	0.0317 (18)	0.056 (2)	0.052 (2)	0.0063 (16)	0.0099 (16)	0.028 (2)
C56	0.0280 (16)	0.0389 (18)	0.0346 (17)	0.0086 (13)	0.0062 (13)	0.0143 (15)
C61	0.0203 (13)	0.0268 (14)	0.0327 (15)	−0.0007 (12)	−0.0045 (10)	0.0073 (14)
C62	0.0336 (17)	0.0422 (18)	0.0351 (17)	−0.0014 (13)	−0.0066 (13)	0.0064 (15)
C63	0.0435 (19)	0.0426 (19)	0.0474 (19)	−0.0066 (17)	−0.0238 (15)	0.0127 (19)
C64	0.0262 (17)	0.0414 (18)	0.079 (3)	−0.0039 (14)	−0.0204 (17)	0.0235 (19)
C65	0.0259 (15)	0.0375 (18)	0.065 (2)	0.0039 (12)	0.0045 (15)	0.0139 (18)
C66	0.0255 (14)	0.0282 (14)	0.0405 (18)	0.0014 (11)	0.0018 (12)	0.0080 (14)
N100	0.064 (2)	0.071 (3)	0.115 (4)	0.008 (2)	−0.011 (2)	0.041 (3)
C100	0.041 (2)	0.072 (3)	0.056 (2)	−0.021 (2)	0.0030 (17)	0.004 (2)
C101	0.039 (2)	0.062 (2)	0.067 (2)	−0.0101 (18)	0.0026 (18)	−0.014 (2)
N200	0.061 (3)	0.065 (2)	0.092 (3)	−0.011 (2)	0.001 (2)	−0.012 (2)
C200	0.047 (3)	0.040 (2)	0.081 (3)	−0.0072 (18)	0.009 (2)	−0.007 (2)
C201	0.079 (4)	0.077 (4)	0.183 (7)	0.013 (3)	−0.035 (4)	−0.006 (4)

### Geometric parameters (Å, °)

**Table:**

Ru1—N2	2.003 (2)	C33—H33	0.95
Ru1—N1	2.016 (2)	C34—C35	1.366 (5)
Ru1—P1	2.3688 (7)	C34—H34	0.95
Ru1—P2	2.3887 (7)	C35—C36	1.393 (5)
Ru1—Cl2	2.4139 (7)	C35—H35	0.95
Ru1—Cl1	2.4308 (7)	C36—H36	0.95
N1—C1	1.135 (3)	C41—C46	1.391 (4)
C1—C2	1.463 (4)	C41—C42	1.401 (4)
C2—H2A	0.98	C42—C43	1.398 (5)
C2—H2B	0.98	C42—H42	0.95
C2—H2C	0.98	C43—C44	1.371 (7)
N2—C3	1.136 (3)	C43—H43	0.95
C3—C4	1.453 (4)	C44—C45	1.380 (7)
C4—H4A	0.98	C44—H44	0.95
C4—H4B	0.98	C45—C46	1.378 (5)
C4—H4C	0.98	C45—H45	0.95
P1—C11	1.831 (3)	C46—H46	0.95
P1—C21	1.835 (3)	C51—C52	1.394 (4)
P1—C31	1.841 (3)	C51—C56	1.404 (4)
P2—C41	1.828 (3)	C52—C53	1.396 (4)
P2—C51	1.834 (3)	C52—H52	0.95
P2—C61	1.841 (3)	C53—C54	1.376 (6)
C11—C16	1.389 (4)	C53—H53	0.95
C11—C12	1.393 (4)	C54—C55	1.374 (6)
C12—C13	1.380 (4)	C54—H54	0.95
C12—H12	0.95	C55—C56	1.391 (4)
C13—C14	1.374 (5)	C55—H55	0.95
C13—H13	0.95	C56—H56	0.95
C14—C15	1.390 (5)	C61—C66	1.382 (4)
C14—H14	0.95	C61—C62	1.404 (4)
C15—C16	1.392 (4)	C62—C63	1.379 (4)
C15—H15	0.95	C62—H62	0.95
C16—H16	0.95	C63—C64	1.375 (5)
C21—C26	1.391 (4)	C63—H63	0.95
C21—C22	1.406 (4)	C64—C65	1.384 (5)
C22—C23	1.389 (4)	C64—H64	0.95
C22—H22	0.95	C65—C66	1.390 (4)
C23—C24	1.386 (5)	C65—H65	0.95
C23—H23	0.95	C66—H66	0.95
C24—C25	1.367 (4)	N100—C100	1.125 (5)
C24—H24	0.95	C100—C101	1.464 (6)
C25—C26	1.394 (4)	C101—H103	0.98
C25—H25	0.95	C101—H102	0.98
C26—H26	0.95	C101—H101	0.98
C31—C36	1.392 (4)	N200—C200	1.114 (5)
C31—C32	1.396 (4)	C200—C201	1.437 (7)
C32—C33	1.393 (4)	C201—H203	0.98
C32—H32	0.95	C201—H202	0.98
C33—C34	1.377 (5)	C201—H201	0.98
			
N2—Ru1—N1	90.03 (9)	C33—C32—H32	119.5
N2—Ru1—P1	90.02 (6)	C31—C32—H32	119.5
N1—Ru1—P1	92.15 (7)	C34—C33—C32	119.7 (3)
N2—Ru1—P2	89.29 (6)	C34—C33—H33	120.1
N1—Ru1—P2	89.55 (7)	C32—C33—H33	120.1
P1—Ru1—P2	178.17 (2)	C35—C34—C33	120.0 (3)
N2—Ru1—Cl2	85.16 (7)	C35—C34—H34	120.0
N1—Ru1—Cl2	175.05 (6)	C33—C34—H34	120.0
P1—Ru1—Cl2	89.02 (2)	C34—C35—C36	121.0 (3)
P2—Ru1—Cl2	89.23 (2)	C34—C35—H35	119.5
N2—Ru1—Cl1	178.93 (7)	C36—C35—H35	119.5
N1—Ru1—Cl1	90.99 (6)	C31—C36—C35	120.1 (3)
P1—Ru1—Cl1	89.59 (3)	C31—C36—H36	120.0
P2—Ru1—Cl1	91.06 (2)	C35—C36—H36	120.0
Cl2—Ru1—Cl1	93.83 (2)	C46—C41—C42	119.3 (3)
C1—N1—Ru1	173.9 (2)	C46—C41—P2	120.5 (2)
N1—C1—C2	177.0 (3)	C42—C41—P2	119.3 (3)
C1—C2—H2A	109.5	C43—C42—C41	119.3 (4)
C1—C2—H2B	109.5	C43—C42—H42	120.3
H2A—C2—H2B	109.5	C41—C42—H42	120.3
C1—C2—H2C	109.5	C44—C43—C42	120.5 (4)
H2A—C2—H2C	109.5	C44—C43—H43	119.8
H2B—C2—H2C	109.5	C42—C43—H43	119.8
C3—N2—Ru1	176.7 (2)	C43—C44—C45	120.2 (4)
N2—C3—C4	178.8 (3)	C43—C44—H44	119.9
C3—C4—H4A	109.5	C45—C44—H44	119.9
C3—C4—H4B	109.5	C46—C45—C44	120.3 (4)
H4A—C4—H4B	109.5	C46—C45—H45	119.8
C3—C4—H4C	109.5	C44—C45—H45	119.8
H4A—C4—H4C	109.5	C45—C46—C41	120.4 (4)
H4B—C4—H4C	109.5	C45—C46—H46	119.8
C11—P1—C21	101.26 (12)	C41—C46—H46	119.8
C11—P1—C31	105.82 (14)	C52—C51—C56	118.5 (3)
C21—P1—C31	99.21 (12)	C52—C51—P2	120.9 (2)
C11—P1—Ru1	111.67 (9)	C56—C51—P2	120.6 (2)
C21—P1—Ru1	120.58 (9)	C51—C52—C53	120.6 (3)
C31—P1—Ru1	116.22 (9)	C51—C52—H52	119.7
C41—P2—C51	103.96 (13)	C53—C52—H52	119.7
C41—P2—C61	103.37 (13)	C54—C53—C52	119.9 (4)
C51—P2—C61	100.04 (12)	C54—C53—H53	120.1
C41—P2—Ru1	109.60 (9)	C52—C53—H53	120.1
C51—P2—Ru1	119.54 (10)	C55—C54—C53	120.5 (3)
C61—P2—Ru1	118.29 (9)	C55—C54—H54	119.8
C16—C11—C12	118.4 (3)	C53—C54—H54	119.8
C16—C11—P1	120.5 (2)	C54—C55—C56	120.4 (3)
C12—C11—P1	120.5 (2)	C54—C55—H55	119.8
C13—C12—C11	120.9 (3)	C56—C55—H55	119.8
C13—C12—H12	119.6	C55—C56—C51	120.1 (3)
C11—C12—H12	119.6	C55—C56—H56	119.9
C14—C13—C12	120.8 (3)	C51—C56—H56	119.9
C14—C13—H13	119.6	C66—C61—C62	118.4 (3)
C12—C13—H13	119.6	C66—C61—P2	119.7 (2)
C13—C14—C15	119.1 (3)	C62—C61—P2	121.8 (2)
C13—C14—H14	120.5	C63—C62—C61	120.4 (3)
C15—C14—H14	120.5	C63—C62—H62	119.8
C14—C15—C16	120.4 (3)	C61—C62—H62	119.8
C14—C15—H15	119.8	C64—C63—C62	120.6 (3)
C16—C15—H15	119.8	C64—C63—H63	119.7
C11—C16—C15	120.4 (3)	C62—C63—H63	119.7
C11—C16—H16	119.8	C63—C64—C65	119.9 (3)
C15—C16—H16	119.8	C63—C64—H64	120.1
C26—C21—C22	119.0 (3)	C65—C64—H64	120.1
C26—C21—P1	122.3 (2)	C64—C65—C66	119.7 (3)
C22—C21—P1	118.8 (2)	C64—C65—H65	120.1
C23—C22—C21	120.4 (3)	C66—C65—H65	120.1
C23—C22—H22	119.8	C61—C66—C65	121.0 (3)
C21—C22—H22	119.8	C61—C66—H66	119.5
C24—C23—C22	120.0 (3)	C65—C66—H66	119.5
C24—C23—H23	120.0	N100—C100—C101	177.2 (5)
C22—C23—H23	120.0	C100—C101—H103	109.5
C25—C24—C23	119.6 (3)	C100—C101—H102	109.5
C25—C24—H24	120.2	H103—C101—H102	109.5
C23—C24—H24	120.2	C100—C101—H101	109.5
C24—C25—C26	121.7 (3)	H103—C101—H101	109.5
C24—C25—H25	119.2	H102—C101—H101	109.5
C26—C25—H25	119.2	N200—C200—C201	179.8 (6)
C21—C26—C25	119.4 (3)	C200—C201—H203	109.5
C21—C26—H26	120.3	C200—C201—H202	109.5
C25—C26—H26	120.3	H203—C201—H202	109.5
C36—C31—C32	118.3 (3)	C200—C201—H201	109.5
C36—C31—P1	125.2 (2)	H203—C201—H201	109.5
C32—C31—P1	116.4 (2)	H202—C201—H201	109.5
C33—C32—C31	120.9 (3)		
			
N2—Ru1—P1—C11	−12.66 (12)	Ru1—P1—C31—C36	−133.4 (2)
N1—Ru1—P1—C11	−102.69 (11)	C11—P1—C31—C32	176.5 (2)
Cl2—Ru1—P1—C11	72.50 (10)	C21—P1—C31—C32	−78.9 (2)
Cl1—Ru1—P1—C11	166.34 (10)	Ru1—P1—C31—C32	52.0 (3)
N2—Ru1—P1—C21	−131.27 (12)	C36—C31—C32—C33	−1.2 (5)
N1—Ru1—P1—C21	138.70 (11)	P1—C31—C32—C33	173.9 (2)
Cl2—Ru1—P1—C21	−46.11 (10)	C31—C32—C33—C34	0.8 (5)
Cl1—Ru1—P1—C21	47.73 (10)	C32—C33—C34—C35	0.8 (5)
N2—Ru1—P1—C31	108.85 (13)	C33—C34—C35—C36	−2.0 (6)
N1—Ru1—P1—C31	18.82 (13)	C32—C31—C36—C35	0.0 (5)
Cl2—Ru1—P1—C31	−165.99 (11)	P1—C31—C36—C35	−174.6 (3)
Cl1—Ru1—P1—C31	−72.16 (11)	C34—C35—C36—C31	1.6 (5)
N2—Ru1—P2—C41	0.84 (12)	C51—P2—C41—C46	147.0 (2)
N1—Ru1—P2—C41	90.87 (12)	C61—P2—C41—C46	42.9 (3)
Cl2—Ru1—P2—C41	−84.33 (11)	Ru1—P2—C41—C46	−84.1 (2)
Cl1—Ru1—P2—C41	−178.15 (11)	C51—P2—C41—C42	−44.1 (3)
N2—Ru1—P2—C51	120.59 (12)	C61—P2—C41—C42	−148.2 (2)
N1—Ru1—P2—C51	−149.38 (12)	Ru1—P2—C41—C42	84.8 (2)
Cl2—Ru1—P2—C51	35.42 (10)	C46—C41—C42—C43	−2.3 (4)
Cl1—Ru1—P2—C51	−58.40 (10)	P2—C41—C42—C43	−171.3 (2)
N2—Ru1—P2—C61	−117.22 (13)	C41—C42—C43—C44	1.6 (5)
N1—Ru1—P2—C61	−27.19 (12)	C42—C43—C44—C45	−0.3 (6)
Cl2—Ru1—P2—C61	157.61 (11)	C43—C44—C45—C46	−0.3 (6)
Cl1—Ru1—P2—C61	63.79 (11)	C44—C45—C46—C41	−0.5 (5)
C21—P1—C11—C16	32.4 (3)	C42—C41—C46—C45	1.8 (4)
C31—P1—C11—C16	135.5 (2)	P2—C41—C46—C45	170.7 (2)
Ru1—P1—C11—C16	−97.1 (2)	C41—P2—C51—C52	142.5 (2)
C21—P1—C11—C12	−156.8 (2)	C61—P2—C51—C52	−110.9 (2)
C31—P1—C11—C12	−53.8 (3)	Ru1—P2—C51—C52	19.9 (3)
Ru1—P1—C11—C12	73.6 (2)	C41—P2—C51—C56	−39.8 (3)
C16—C11—C12—C13	−1.4 (4)	C61—P2—C51—C56	66.8 (3)
P1—C11—C12—C13	−172.3 (2)	Ru1—P2—C51—C56	−162.40 (19)
C11—C12—C13—C14	0.5 (5)	C56—C51—C52—C53	0.0 (4)
C12—C13—C14—C15	0.4 (5)	P2—C51—C52—C53	177.7 (2)
C13—C14—C15—C16	−0.4 (5)	C51—C52—C53—C54	−0.6 (5)
C12—C11—C16—C15	1.4 (5)	C52—C53—C54—C55	0.6 (5)
P1—C11—C16—C15	172.3 (3)	C53—C54—C55—C56	−0.1 (5)
C14—C15—C16—C11	−0.5 (5)	C54—C55—C56—C51	−0.5 (5)
C11—P1—C21—C26	−125.0 (2)	C52—C51—C56—C55	0.5 (4)
C31—P1—C21—C26	126.7 (2)	P2—C51—C56—C55	−177.2 (2)
Ru1—P1—C21—C26	−1.3 (3)	C41—P2—C61—C66	−150.7 (2)
C11—P1—C21—C22	54.7 (2)	C51—P2—C61—C66	102.2 (2)
C31—P1—C21—C22	−53.6 (2)	Ru1—P2—C61—C66	−29.4 (3)
Ru1—P1—C21—C22	178.40 (18)	C41—P2—C61—C62	32.4 (3)
C26—C21—C22—C23	1.2 (4)	C51—P2—C61—C62	−74.7 (3)
P1—C21—C22—C23	−178.5 (2)	Ru1—P2—C61—C62	153.7 (2)
C21—C22—C23—C24	−0.6 (5)	C66—C61—C62—C63	0.0 (5)
C22—C23—C24—C25	0.3 (5)	P2—C61—C62—C63	176.9 (2)
C23—C24—C25—C26	−0.5 (5)	C61—C62—C63—C64	1.4 (5)
C22—C21—C26—C25	−1.3 (4)	C62—C63—C64—C65	−1.5 (5)
P1—C21—C26—C25	178.3 (2)	C63—C64—C65—C66	0.2 (5)
C24—C25—C26—C21	1.0 (5)	C62—C61—C66—C65	−1.3 (4)
C11—P1—C31—C36	−8.8 (3)	P2—C61—C66—C65	−178.3 (2)
C21—P1—C31—C36	95.7 (3)	C64—C65—C66—C61	1.2 (5)

### Hydrogen-bond geometry (Å, °)

**Table:**

*D*—H···*A*	*D*—H	H···*A*	*D*···*A*	*D*—H···*A*
C4—H4B···Cl1^i^	0.98	2.68	3.560 (3)	149
C101—H101···Cl1^ii^	0.98	2.80	3.698 (4)	153
C2—H2C···Cl2^iii^	0.98	2.57	3.544 (3)	175
C101—H102···Cl2	0.98	2.62	3.554 (4)	158
C2—H2A···N100^i^	0.98	2.60	3.519 (5)	155
C101—H103···N200	0.98	2.72	3.645 (6)	158
C201—H201···N200^iv^	0.98	2.66	3.526 (7)	148
C64—H64···Cg1^iii^	0.95	2.96	3.715 (4)	138

Symmetry codes: (i) −*x*+2, *y*+1/2, −*z*+1/2; (ii) *x*−1, *y*, *z*; (iii) *x*+1, *y*, *z*; (iv) *x*−1/2, −*y*+1/2, −*z*.
